# Arbuscular Mycorrhiza Changes the Impact of Potato Virus Y on Growth and Stress Tolerance of *Solanum tuberosum* L. *in vitro*

**DOI:** 10.3389/fmicb.2019.02971

**Published:** 2020-01-15

**Authors:** Edyta Deja-Sikora, Anita Kowalczyk, Alina Trejgell, Adriana Szmidt-Jaworska, Christel Baum, Louis Mercy, Katarzyna Hrynkiewicz

**Affiliations:** ^1^Department of Microbiology, Faculty of Biological and Veterinary Sciences, Nicolaus Copernicus University, Toruń, Poland; ^2^Centre for Modern Interdisciplinary Technologies, Nicolaus Copernicus University, Toruń, Poland; ^3^Chair of Plant Physiology and Biotechnology, Faculty of Biological and Veterinary Sciences, Nicolaus Copernicus University, Toruń, Poland; ^4^Chair of Soil Science, Faculty of Agricultural and Environmental Sciences, University of Rostock, Rostock, Germany; ^5^INOQ GmbH, Schnega, Germany

**Keywords:** *Solanum tuberosum* L., potato virus Y (PVY), *Rhizophagus irregularis*, arbuscular mycorrhiza, oxidative stress

## Abstract

Under the field conditions crop plants interact with diverse microorganisms. These include beneficial (symbiotic) and phytopathogenic microorganisms, which jointly affect growth and productivity of the plants. In last decades, production of potato (*Solanum tuberosum* L.) suffers from increased incidence of potato virus Y (PVY), which is one of most important potato pests. Arbuscular mycorrhizal fungi (AMF) are common symbionts of potato, however the impact of mycorrhizal symbiosis on the progression of PVY-induced disease is scarcely known. Therefore, in the present study we investigated the effect of joint PVY infection and mycorrhizal colonization by *Rhizophagus irregularis* on growth traits of the host potato plant (cv. Pirol). The tested PVY isolate belonged to N-Wilga strain group, which is considered to be predominant in Europe and many other parts of the world. The viral particles were concentrated in the leaves, but decreased the root growth. Furthermore, the infection with PVY evoked prolonged oxidative stress reflected by increased level of endogenous H_2_O_2_. AMF alleviated oxidative stress in PVY-infected host plants by a substantial decrease in the level of shoot- and root-derived H_2_O_2_, but still caused asymptomatic growth depression. It was assumed that mycorrhizal symbiosis of potato might mask infection by PVY in field observations.

## Introduction

Plant growth and physiology are affected by both symbionts and phytopathogens co-infecting the same host. These tripartite biotic interactions (involving antagonistic, protective, exclusive, or synergistic effects) are of particular interest with regard to crop plants, since they strongly influence the crop productivity. It is documented that under the field conditions the majority of crop plants establishes symbiotic association between their roots and arbuscular mycorrhizal fungi (AMF) being an inherent component of each agricultural ecosystem (Smith and Smith, 2011; Van Geel et al., 2016). In this endomycorrhizal relationship both partners benefit from one another. AMF hyphae act as root system extension and explore the soil outside the rhizosphere. Host plant, due to high absorptive capacity of extraradical mycelium, gains an easier acquisition of soil water and slowly diffusing mineral compounds (in particular phosphorus and nitrogen ions), which results in the improved plant nutritional status and fitness (Bitterlich and Franken, 2016). In exchange, plants furnish a habitat (as physical and favorable physiological support) that allows AMF to uptake energy (i.e., carbohydrates and lipids) in order to complete their life cycle (Mercy et al., 2017; Rich et al., 2017; Wang et al., 2017). Furthermore, mycorrhizal plants often display enhanced tolerance to abiotic stress factors (e.g., drought or salinity) and increased resistance to both phytopathogen attack and development of phytopathogen-induced disease (Bücking et al., 2016; Deja-Sikora et al., 2019). These nutritional and non-nutritional (bioprotective) benefits of endomycorrhiza contribute to the improved crop yields and encourage the wide application of AMF-based natural biofertilizers to support the sustainable agriculture systems (Hart et al., 2015; Rouphael et al., 2015; Basu et al., 2018; Bitterlich et al., 2018). Recently, AMF are even perceived a key factor for optimization of crop productivity, especially in the low-input agriculture (Verbruggen et al., 2013).

Potato (*Solanum tuberosum* L.) belongs to the most meaningful horticultural species grown worldwide for food and industrial purposes. Numerous experiments conducted under greenhouse, shade house and field conditions showed that potato roots are prone to establish endomycorrhiza with several AMF species, including *Rhizophagus intraradices* (formerly *Glomus intraradices*), *Rhizophagus irregularis* (formerly *Glomus irregulare*), *Funneliformis mosseae* (formerly *Glomus mosseae*), or *Gigaspora* sp. (Douds et al., 2007; Gallou et al., 2011; Lone et al., 2015; Hijri, 2016). Mycorrhizal potato plants were reported to display improved growth, pathogen resistance, and productivity compared to non-mycorrhizal ones (Douds et al., 2007; Bharadwaj et al., 2008). The results of large scale field trials indicated that inoculation of potato with *R. irregularis* DAOM 197198 caused significant increase in tuber yield, and the effect was cultivar independent (Hijri, 2016). The positive effect of *R. intraradices* and *F. mosseae* on the host morphological parameters was found for two potato cultivars (Jyoti and TPS) (Lone et al., 2015). Root colonization with AMF improved fresh and dry matter of both plant shoot and root, increased the chlorophyll content and tuber yield. This observation was in line with the other study that demonstrated the enhancement of potato (cv. Yungay) growth parameters upon mycorrhization with *R. intraradices* due to greater uptake of P, Fe, and Mg as well as higher efficiency of P utilization (Davies et al., 2005). Furthermore, colonization with AMF was linked to the lower incidence of infection with some potato pathogens or reduced disease severity. *G. etunicatum* and *R. intraradices* were associated with milder symptoms of *Rhizoctonia solani*-induced disease in potato (cv. Goldrush) (Yao et al., 2002). AMF were indicated to have bio-protective function against leaf pathogen *Phytophthora infestans*, as mycorrhizal potato plants (cv. Bintje) showed decreased progress of disease resulting from activation of plant systemic resistance to pathogen attack (Gallou et al., 2011). Nevertheless, the results of investigations on the bio-control of potato viruses by endomycorrhiza are less conclusive.

Potato virus Y (PVY) is an extremely devastating pathogen of *S. tuberosum* that dramatically reduces tuber yield and quality causing huge economical losses worldwide (Funke et al., 2016). PVY causes foliar and/or tuber disease with variable symptoms depending on virus strain, host growth stage and susceptibility, and environmental conditions (Fox et al., 2017). Currently, PVY^N^ and recombinant PVY^NTN^ and PVY^N–Wi^ strains largely predominate under field conditions, accounting for > 90% of all PVY cases (Davie et al., 2017). PVY is transmitted non-persistently by aphids (e.g., *Myzus persicae*) being so far the only identified vectors for this pathogen. However, the application of insecticides seems to be ineffective in PVY infection control. New, potentially successful strategies to manage the virus may involve the utilization of microbiological (biocontrol) agents comprising bacterial and fungal species (Al-Ani et al., 2013). These plant-associated microorganisms can alleviate the negative impact of virus, e.g., by modulating the level of plant stress response. The treatment of potato tubers with either *Pseudomonas fluorescens* or *Rhodotorula* sp. was found to reduce the severity of PVY-induced disease (Al-Ani et al., 2013). Unfortunately, the interaction between PVY and AMF (known for their bio-protective function against different potato pathogens) is poorly characterized. The worsening of growth parameters in *R. irregularis*-inoculated potato plants along with an increase in the symptoms of PVY-induced disease were previously showed in the pot experiment (Sipahioglu et al., 2009). However, no data for potato plants grown *in vitro* are available.

Plants induce H_2_O_2_ signaling in response to both pathogen attack and symbiosis establishment, e.g., during initial stage of endomycorrhiza development (Pozo and Azcon-Aguilar, 2007; Nath et al., 2016). The specific plant-AMF interaction resulting in H_2_O_2_ synthesis was previous indicated in several articles (Puppo et al., 2013; Nath et al., 2016; Kapoor and Singh, 2017), which can be related to the temporal and spatial control of plant root colonization (Salzer et al., 1999). It was indicated that H_2_O_2_ accumulated in root cortical cells, in the vicinity of arbuscules, as well as around the intraradical hyphal tips penetrating the host cells (Salzer et al., 1999). Furthermore, H_2_O_2_ was reported to act as long-distance signal molecule for activation of biotic stress adaptation mechanisms (Sewelam et al., 2016). Among reactive oxygen species (ROS), membrane-permeable H_2_O_2_ is thought to be key player involved in regulation of many biological reactions, e.g., stress response (Saxena et al., 2016).

The goal of this investigation was to examine the effect of (i) PVY infection, (ii) AMF inoculation, and (iii) PVY-AMF co-infection on both vegetative growth parameters and stress response in potato host plants grown *in vitro*. Since plant cells regulate oxidative metabolism in response to pathogen attack we analyzed the level of hydrogen peroxide (H_2_O_2_) in shoots and roots of PVY-infected mycorrhizal plants. We hypothesized that *R. irregularis* can improve the growth of PVY-infected potato plants by alleviating the negative impact of virus. By verification of this hypothesis we wanted to check the role of AMF in biocontrol of PVY.

## Materials and Methods

### Biological Material

Potato virus Y-infected and virus-free plantlets of *S. tuberosum* cvs. Pirol, Delikat and Schubert were *in vitro* subcultured on the standard Murashige and Skoog (MS) medium without growth regulators (pH 5.8). Single-node cuttings were aseptically transferred into MS medium (Duchefa Biochemie, Haarlem, Netherlands) supplemented with 30 g l^–1^ sucrose and solidified with 7 g l^–1^ agar (Sigma-Aldrich, St. Louis, MO, United States). The plants were cultured in growth chamber under the continuous white fluorescent light (45 μmol m^–2^ s^–1^) at 26°C ± 1°C.

Virus-positive *in vitro* plantlets used in this study were infected with the same strain of PVY before the experiment was started. Inoculation with PVY was done with carborundum (silicon carbide) method using 2-week plantlets as recipients. Leaf of donor PVY-positive potato plant was homogenized in cold 10 mM potassium phosphate buffer pH 7.0 (ratio 1:10) using sterilized mortar and pestle. The homogenate was gently rubbed with swab into the recipient leaves, that were previously dusted with 600-mesh carborundum. The inoculated plants were cultured for 3 weeks at 26°C. The infection was checked with PVY-AgriStrip tests (Bioreba AG, Reinach, Switzerland). Virus-infected plantlets were *in vitro* subcultured for several weeks to confirm the stable infection.

*Rhizophagus irregularis* line QS81 (provided by INOQ, GmbH, Schnega, Germany) was cultured on dual-compartment plates using Ri T-DNA transformed carrot roots (*Daucus carota* L.) as a host for the fungus. Both AMF and roots were grown at 23–25°C on the modified Strullu and Romand (MSR) medium solidified with 3 g l^–1^ Gelrite (Duchefa Biochemie, Haarlem, Netherlands). MSR lacked sucrose and vitamins in the fungus compartment.

### Experimental Design

**FIGURE 1:**
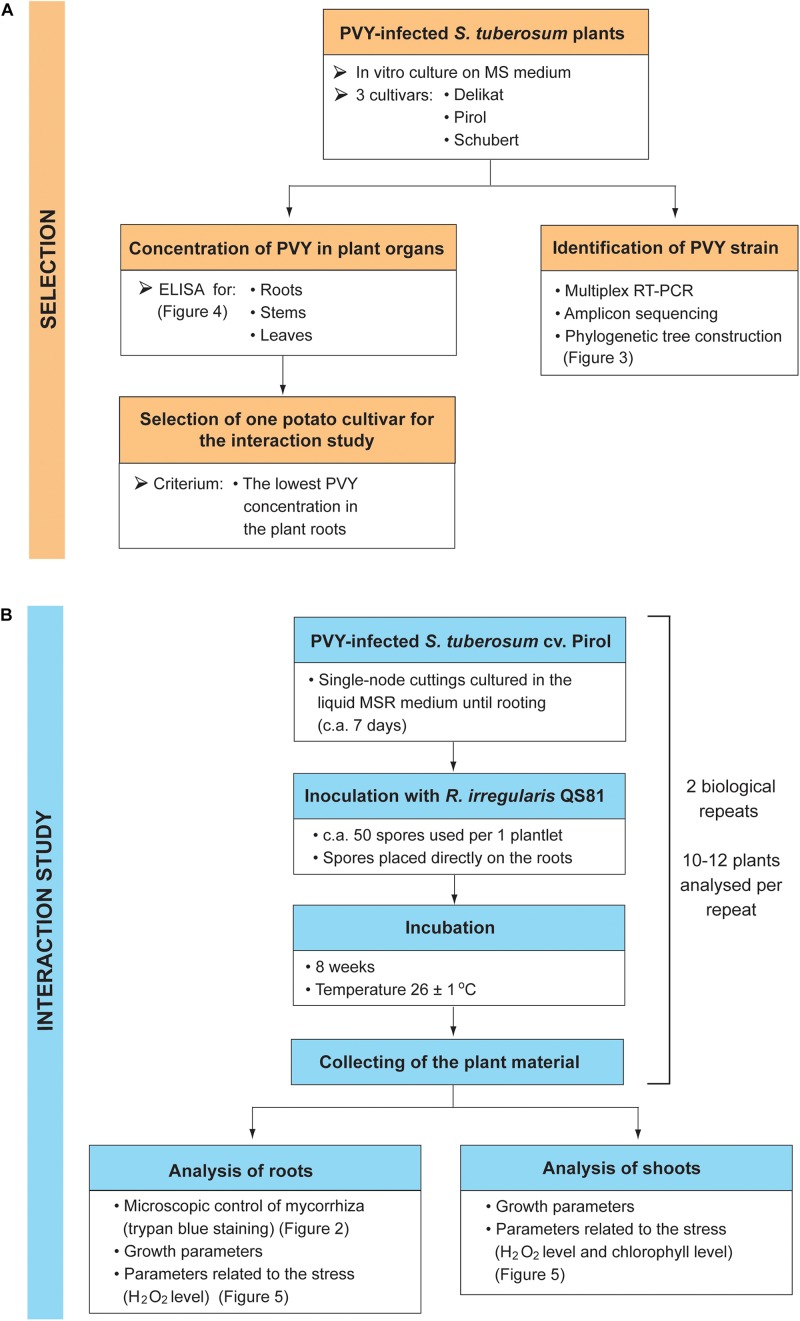
Design of the experiment. The study consisted of two stages: the selection of potato cultivar based on the PVY concentration in the plant roots **(A)**, and the examination of AMF effect on the growth and stress response of PVY-infected plants **(B)**.

The presented study consisted of two stages: the selection of potato cultivar having the lowest concentration of PVY in the plant roots (Figure 1A), and the examination of AMF effect on the growth and stress response of PVY-infected plants (Figure 1B).

### PVY Strain Identification

Potato virus Y strain in virus infected potato plantlets was identified using multiplex reverse-transcriptase PCR (RT-PCR) assay as described by Lorenzen et al. (2006). The protocol was based on the usage of 12-primer set targeted at specific recombination junctions within PVY genomes to discriminate between different strains.

Total RNA from plant tissue was isolated using RNA Extracol Reagent (EURx, Gdańsk, Poland). DNase I-treated RNA samples were reverse transcribed into cDNA using smART Reverse Transcriptase Kit (EURx, Gdańsk, Poland) with random hexamers. Multiplex RT-PCR for PVY identification was performed according to Lorenzen’s protocol. Amplicons were analyzed on 2% agarose gel and sequenced with Sanger method using BigDye Terminator v3.1 Cycle Sequencing Kit (Thermo Fisher Scientific, Waltham, MA, United States). Sequencing reactions were analyzed with ABI3730 Genetic Analyzer (Oligo IBB PAS, Warsaw, Poland). Reads were quality checked with MEGA X software and sequences were deposited in GenBank under accession numbers MK455818 and MK455819. The phylogenetic tree for reference PVY strains (according to Glais et al., 2017) was generated with ML algorithm in MEGA X (Kumar et al., 2018).

### TAS-ELISA for PVY Concentration

Potato virus Y concentration in virus infected potato cultivars was examined with TAS-ELISA using ELISA Reagent Set for Potato virus Y (Agdia, Inc., Elkhart, IN, United States). For each of three biological repeats, leaves, stems and roots were collected separately from three 4-week old plantlets (per cultivar) grown *in vitro* under conditions described above. 30–50 mg of fresh plant tissue was homogenized in general extract buffer (GEB) at a 1:10 ratio as recommended by the manufacturer. The assay was performed according to the manufacturer‘s protocol. Positive controls for potato virus Y (Agdia, Inc., Elkhart, IN, United States) were processed along with the analyzed samples to validate the measurements. Three virus-free plantlets of each cultivar were included as negative controls. The buffer wells were prepared to subtract the background absorbance. The sample was regarded PVY infected if its absorbance value was greater than three-times the average value of negative control. PVY concentration in the sample was calculated in relation to positive control.

### Inoculation of Potato Plantlets With AMF

**FIGURE 2:**
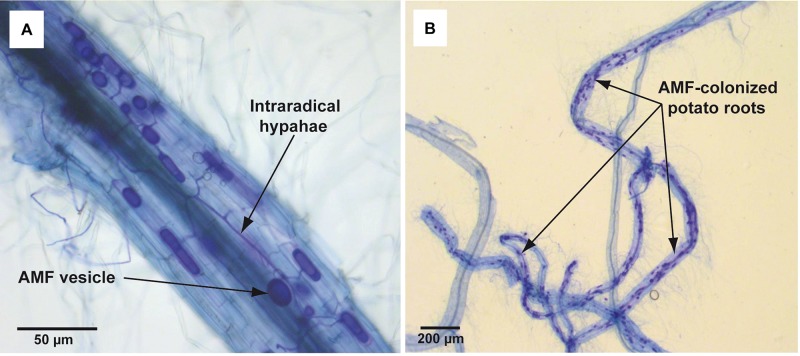
Trypan blue-stained mycorrhizal structures in potato roots cv. Pirol colonized with *Rhizophagus irregularis*. The mycorrhization was performed in liquid *in vitro* system and the microscopic analysis was done in 8 week post-inoculation. Panel **(A)** shows dark blue-stained AMF structures (vesicles and intraradical hyphae) inside the potato root (100x magnification). Panel **(B)** shows dark blue-stained fragments of potato roots strongly colonized with AMF (12.5x magnification).

In total 15 single-node cuttings were transferred to glass tubes (ø 25 mm) with 10 ml of liquid (non-solidified) MSR medium without plant regulators. Tubes contained also 1.5 g of perlite in order to maintain the shoot in a vertical position. After 6–7 days of culturing (26 ± 1°C, 16/8 h L/D), when adventitious roots were observed (c.a. 10 mm), plantlets were inoculated with spores of *R. irregularis.* The pieces of solid MSR medium containing c.a. 50 spores were placed directly on the emerging roots. Then the lower parts of the tubes were covered with aluminum foil to prevent the light access. The plantlets were maintained for 8 weeks under conditions described above. Mycorrhiza development in roots was confirmed at the end of incubation period by standard Trypan blue staining (Phillips and Hayman, 1970) and microscopic analysis (Figure 2). The experiment was performed in duplicate (two biological repeats). Each replicate consisted of 10–12 plants (technical repeats), that were selected for the parameters examination.

### Plant Growth Parameters and the Measurement of H_2_O_2_ Level

Eight-week-old potato plants were removed from the experimental medium and the following parameters were measured: shoot height, number of nodes, root length, and root and shoot fresh weight.

Total content of chlorophyll in fresh leaves (expressed in μg g^–1^ FW) was determined using the method by Lichtenthaler and Buschmann (2001). Photosynthetic pigments including chlorophyll were extracted from 20 mg of homogenized tissue by incubating in 10 ml of 95% (v/v) ethanol for 72 h under dark conditions at 4°C. The absorbance of the supernatant was measured at wavelengths of 664.2 nm (A_664_._2_) and 648.6 nm (A_648_._6_) using UV-VIS Spectrophotometer UV-1601PC (Shimadzu, Kyoto, Japan). All measurements were performed in triplicate. Total chlorophyll content was calculated from the following formula: total volume of chlorophyll = 5,24^∗^(A_664.2_) + 22,24^∗^(A_648.6_).

H_2_O_2_ level in plant tissue (root and shoot) was measured with colorimetric method using potassium iodide (Velikova et al., 2000). Briefly, 100 mg of lyophilized plant tissue powder was treated with 1 ml of 0.1% trichloroacetic acid (TCA) and incubated on ice for 20 min with agitation. The homogenate was centrifuged (10,000 × *g*; 10 min, 4°C) and 0.5 ml of supernatant was added to 0.5 ml of 10 mM phosphate buffer (pH 7.0) and 1 ml of 1M potassium iodide. The mixture was incubated in darkness for 1 h at room temperature. The absorbance was measured at 390 nm. The samples were measured in triplicate against standard curve. The concentration of H_2_O_2_ was calculated from the equation: C_H__2__O__2_ = (C_total_^∗^ V_total_)(V * w); C_total_ – nanomolar concentration of H_2_O_2_ determined from standard curve; V_total_ – total volume of supernatant (1 ml); V – volume of supernatant in the reaction mixture (0.5 ml); w – sample weighting.

### Statistical Analyses

Observations lying beyond 75th percentile (outliers) were detected using Outliers package in R and removed from dataset. Normality of data distribution was checked with Shapiro–Wilk *W*-test. Levene’s test was used to check the homogeneity of variance. The results of the experiment were analyzed using Student’s *t*-test (for equal variances) or Welch’s *t*-test (for unequal variances) to evaluate the differences in studied parameters between control (non-inoculated) and mycorrhizal potato plants. Two-way ANOVA was calculated to examine the effect of AMF-inoculation on the parameters of virus-free (healthy) and PVY-infected plants. Statistical analyses were performed in Statistica 7.0 (StatSoft, Inc., Tulsa, OK, United States).

## Results

### PVY Identification and Distribution in Plant Tissues

**FIGURE 3:**
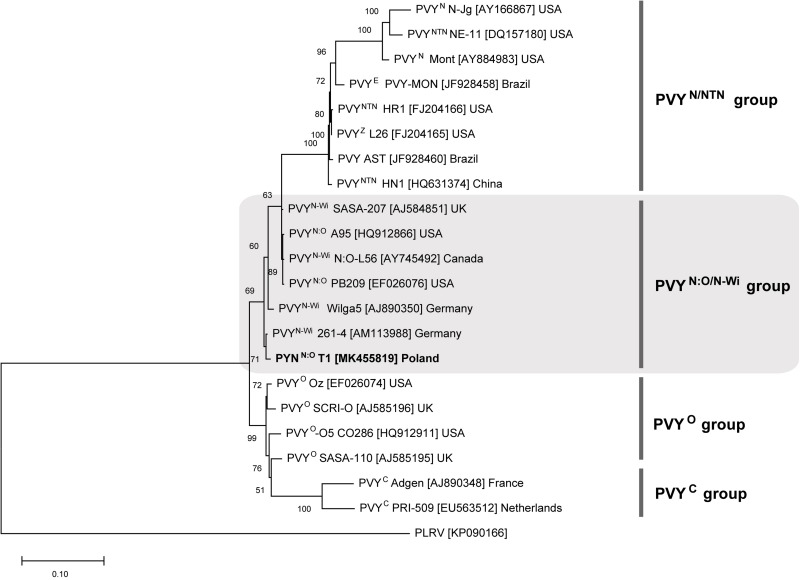
Phylogenetic analysis of reference PVY isolates belonging to different strain groups. The tree was generated with ML method. PVY isolate identified in this study (PVY^N:O^-T1) is most closely related to PVY^N–Wi^ (N-Wilga) strain group (shaded). Potato leafroll virus (PLRV) was used as an outgroup.

Multiplex RT-PCR assay revealed that analyzed potato cultivars, i.e., Pirol, Delikat and Schubert, were infected with N:O/N-Wi type A recombinant variant of PVY. Sequencing of PVY genome fragments containing specific recombination junctions confirmed the result of PCR assay. Identified PVY strain, denoted as PVY^N:O^-T1, was closely related to PVY^N–Wi^ (N-Wilga) and PVY^N:O^ genotypes (above 99% of similarity). PVY^N:O^-T1 was placed within PVY^N:O/N–Wi^ group in the phylogenetic tree (Figure 3). Potato cultivars infected with PVY^N:O^-T1 were asymptomatic, since no foliar disease was noticed.

**FIGURE 4:**
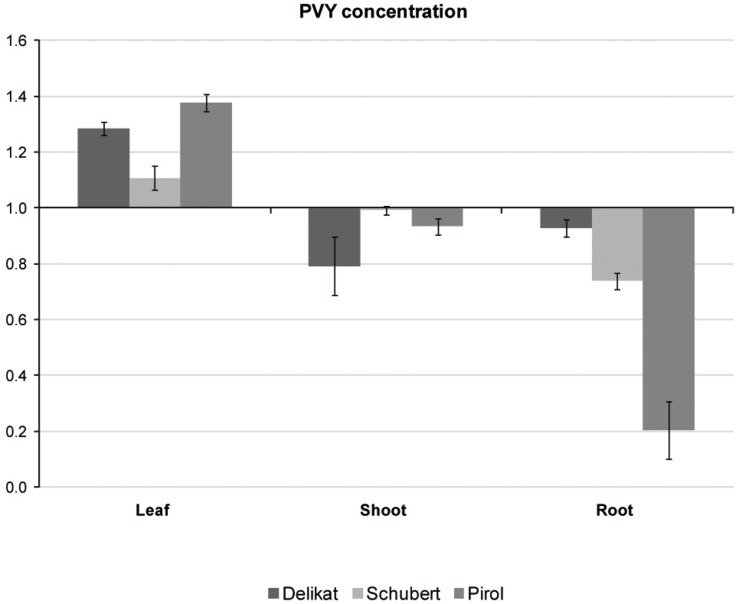
Relative concentrations of PVY measured with TAS-ELISA in different organs (leaves, shoots, and roots) of three PVY-positive potato cultivars, i.e., Pirol, Delikat and Schubert. The values are expressed as relative to commercial positive control for PVY representing the axis of value 1.

Potato virus Y distribution in different organs of virus-positive potato plantlets grown *in vitro* was examined with TAS-ELISA method. The analysis indicated that all tested cultivars were systemically infected with PVY (Figure 4). Irrespectively of potato cultivar, virus preferentially accumulated in leaves. Decreasing concentration of PVY was detected in stems, however in Schubert and Pirol the lowest titer of virus was observed in roots. Due to the highest difference between leaf and root PVY concentration in Pirol (ratio 6.7 compared to 1.5 in Schubert and 1.4 in Delikat), the roots of this potato cultivar could be least impacted by the virus. Since physiological condition of the root is essential for successful establishment of mycorrhiza, *S. tuberosum* cv. Pirol was chosen for studying the interaction between PVY and *R. irregularis* in tripartite (host plant-PVY-AMF) biosystem.

### The Effect of PVY on Plantlet Growth Parameters and H_2_O_2_ Level

Although undetectable by visual inspection, PVY^N:O^-T1 isolate noticeably changed growth parameters and H_2_O_2_ level in potato cv. Pirol. The average length of shoot significantly decreased in PVY-positive plantlets compared to virus-free (control) ones (by above 25%; from 112.6 to 83.8 mm) (Supplementary Table 1). Virus-caused reduction in average length of root was even more dramatic reaching nearly 68% (from 87.4 to 27.6 mm). Furthermore, PVY infection significantly influenced the host biomass. The virus provoked a decrease in fresh weight of shoot (by 25%; from 172.8 to 129 mg) and root (by nearly 61%, from 47.8 to 18.7 mg), resulting in total plantlet biomass reduction by 33%. Moreover, PVY significantly affected H_2_O_2_ level in host tissues. H_2_O_2_ concentration was 1.9-fold higher in shoots (0.81 vs. 1.51 μmol g^–1^ FW) and 3.1-fold higher in roots (0.87 vs. 2.73 μmol g^–1^ FW) of virus-positive plantlets compared to the control ones (Supplementary Table 2). The virus exerted the influence neither on the number of nodes that invariably was 8 (data not shown) nor the content of chlorophyll.

### The Effect of *R. irregularis* on Growth Parameters and H_2_O_2_ Level in Healthy and PVY-Infected Plantlets

**FIGURE 5:**
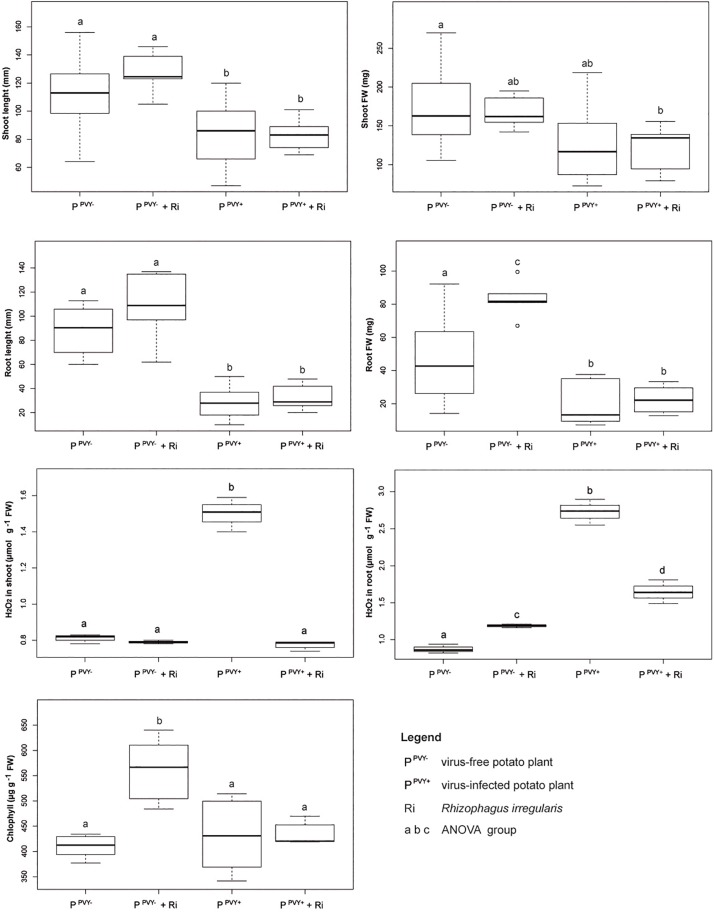
The impact of *R. irregularis* inoculation on growth parameters (shoot length and fresh weight, root length and fresh weight), stress response (H_2_O_2_ level in shoot and root) and chlorophyll content in virus-free and PVY-infected plantlets of potato cv. Pirol.

Inoculation of potato cv. Pirol with *R. irregularis* seemed to have no effect on the length of shoot and root, as well as fresh weight of shoot, irrespectively of PVY infection (Figure 5). The roots colonized by *R. irregularis* were noticeably longer in both the virus-free (87.4 mm in control vs. 107.2 mm after inoculation) and the virus-positive plantlets (27.6 vs. 32.1 mm), however no statistical significance of this result was found (Supplementary Table 1). Furthermore, *R. irregularis* significantly increased the root biomass of healthy plantlets (by nearly 74%; 47.8 vs. 83 mg), but the fungus exerted no influence on the roots of PVY-infected ones. Similar result was observed for chlorophyll content that was considerably higher after inoculation, but only in leaves of virus-free hosts (407 vs. 564 μg g^–1^ FW). The presence of PVY masked the effect of mycorrhiza and the chlorophyll content was at the level of control plants (Supplementary Table 2). Colonization by *R. irregularis* exerted the strongest effect on the level of H_2_O_2_ in host tissues. In the absence of PVY infection the level of H_2_O_2_ in plant shoot remained unchanged upon mycorrhization, however the level of H_2_O_2_ in plant root was significantly raised (from 0.87 to 1.19 μmol g^–1^ FW). The result was different in the PVY-infected plants, because the interaction with *R. irregularis* induced dramatic decrease in H_2_O_2_ level in plantlet shoot (by 48%; from 1.51 to 0.78 μmol g^–1^ FW) and root (by 39.5%; from 2.73 to 1.65 μmol g^–1^ FW).

## Discussion

Virus-positive potato cultivars used in this study, i.e., Pirol, Delikat and Schubert, were systemically infected with N:O recombinant variants of PVY. Such mosaic genotypes phylogenetically originate from an ancestral genome consisting of fragments exchanged between PVY^N^ (necrotic) and PVY^O^ (ordinary) strain (Lorenzen et al., 2006; Karasev et al., 2011). Monitoring of PVY strain incidence conducted during last decades showed the common shift from PVY^O^ strains towards recombinant ones (Crosslin et al., 2006; Davie et al., 2017). This trend is observed worldwide and according to current estimation recombinant PVY^N^ strains may account for up to 90% of all PVY cases found under field conditions (Davie et al., 2017). Based on sequence similarity analysis our PVY isolate (denoted as PVY^N:O^-T1) was identified to be closely related to PVY^N–Wi^ (N-Wilga) strain group. Wide distribution of PVY^N–Wi^ strains becomes more and more apparent nowadays (Visser et al., 2012; Quenouille et al., 2013; Green et al., 2017). The study by Yin et al. (2012) demonstrated that recombinant PVY belonging to the PVY^N–Wi^ along with PVY^NTN^ are predominant variants of the virus infecting potato crops in Poland. Similar findings were previously reported for the other parts of the world, e.g., the United States, Canada and potato-producing regions in South Africa (Crosslin et al., 2006; Visser and Bellstedt, 2009; Gray et al., 2010). Although the members of PVY^N:O/N–Wi^ share the properties of both parental strains, they tend to induce barely detectable disease that due to mild symptoms remains unnoticed during visual inspection (Gray et al., 2010; Funke et al., 2016; Glais et al., 2017). Furthermore, these strains can also remain latent (symptomless) in potato plants (Glais et al., 2005; Kamangar et al., 2014), which is in line with results of our studies. *S. tuberosum* L. plantlets cvs. Pirol, Delikat and Schubert infected with PVY^N:O^-T1 were asymptomatic under *in vitro* conditions, however the virus was detectable in all examined organs, i.e., roots, stems, and leaves. Since viral infection caused neither foliar nor tuber disease it can be concluded that host-PVY interaction was compatible, and tested cultivars were susceptible but tolerant to these PVY isolates. Measured concentrations of PVY^N:O^-T1 differed across the studied cultivars and plant organs. It is not surprising as PVY isolates, in spite of close phylogenetic relatedness, may behave in a contrasting way and accumulate to different level in the same host cultivar (Davie et al., 2017). The highest titer of PVY was found in the leaves of potato plantlets, irrespectively of analyzed cultivar. Our result is partially in opposition to the study by Kogovšek et al. (2011) showing different distribution of PVY^NTN^ strain within potato plants maintained in growth chamber. The authors reported high accumulation of the virus in symptomatic leaves and stems of sensitive potato cv. Igor, while virus amount in asymptomatic leaves was low or even undetectable. Nevertheless, similarly to Kogovšek et al. (2011) we also found the lowest concentration of PVY in roots of two tolerant potato cultivars (Pirol and Schubert). This partial discrepancies in results can be explained by variable distribution pattern of different PVY strains depending on individual virus characteristics, cultivar susceptibility and specific environmental conditions. Our observation can be also supported by results described by Mehle et al. (2004) indicating different kinetics of PVY multiplication and accumulation in organs of sensitive, tolerant and resistant potato cultivars.

Our study demonstrated that infection with PVY^N:O^-T1, although symptomless, inhibited the vegetative growth of tolerant potato cv. Pirol, causing reduction in plantlet total biomass by 33%, which was explicitly visible by root and shoot length shortening. PVY infection was previously found to be associated with axillary growth retardation (measured as shoot length decrease) in potato plantlets cvs. Desirée, Igor and Pentland Squire maintained under *in vitro* culture conditions (Anžlovar et al., 1996). However, the effect was more pronounced in sensitive cultivars (Desirée and Igor) than tolerant one (Pentland Squire). Additionally, Anžlovar et al. (1996) showed that virus exerted no influence on the number of nodes. This observation is in agreement with the results of our study, since we found invariable number of nodes in control and PVY-positive plantlets. We demonstrated that the presence of PVY most negatively impacted the development of plantlet roots, causing dramatic decrease in their length (by c.a. 68%) and fresh weight (by c.a. 61%). Similarly, Dolenc and Dermastia (1999) indicated that PVY strongly reduced growth capacity of primary and secondary roots in potato cv. Igor, due to pronounced histological changes in the root apical meristems. Within last years, adverse effect of PVY infection on growth parameters (including shoot and root length) of Chinese potato cv. Zihuabai was reported by Li et al. (2013).

Apart from changed growth parameters, potato cv. Pirol infected with PVY^N:O^-T1 displayed also strongly elevated endogenous level of H_2_O_2_. Additionally, comparison of H_2_O_2_ concentration in plantlet shoot and root, showed that the second one was more severely impacted by the virus. Previously, Thiem et al. (2014), using the same potato cv. Pirol grown in pots, found the long-term increase in amount of endogenous H_2_O_2_ to be associated with PVY presence. Furthermore, accumulation of H_2_O_2_ in response to viral infection was also shown for other host plant and pathogen (i.e., tobacco and M strain of Cucumber mosaic virus, M-CMV) (Lei et al., 2016). According to literature data, oxidative metabolism, involving utilization of ROS as signal factors, is associated with plant defense response to the pathogen invasion (Shetty et al., 2008; Saxena et al., 2016; Gonzalez-Bosch, 2018). H_2_O_2_ may play a pivotal roles in pathogen control comprising (i) induction of the oxidative burst in hypersensitive response (HR) in order to inhibit pathogen infection development and (ii) activation of biotic stress response mechanism, e.g., SAR (systemic acquired resistance) pathway (Gilroy et al., 2016; Hernández et al., 2016). On the other side, constantly maintained high concentration of endogenous H_2_O_2_ may exert toxic effect on plant development and contribute to the biomass reduction (Gapper and Dolan, 2006), which is suggested in this and previous studies (Thiem et al., 2014).

Current study examined the interaction between PVY and *R. irregularis* sharing the same host plant. The knowledge on the way how pathogen-symbiont interplay shape the host plant condition is still scarce. Plant root growth capacity is essential for successful establishment of mycorrhiza. Therefore, we used potato cv. Pirol (having the lowest concentration of PVY in the roots) to minimize negative effect of the virus on the symbiosis development. We noticed that healthy plantlets colonized with *R. irregularis* displayed growth parameters similar to the control, with the only significant differences found in the higher root biomass (but not length) and higher chlorophyll content upon mycorrhization. Beneficial effect of AMF on root biomass production was already described for potato (Davies et al., 2005; Thiem et al., 2014) as well as for the other plant species (Saia et al., 2015; Chen M. et al., 2017; Jacott et al., 2017; Shao et al., 2018). We cannot exclude that enhanced biomass (but not the length) of root system could be associated with some structural changes that are known to be induced by AMF (e.g., enlargement of root cortex due to an extra cell layer development for accommodation of fungal structures) (Dreyer et al., 2014). However, it is even more probable that improved nutritional status of *R. irregularis-*inoculated potato cv. Pirol, due to more efficient acquisition of water and nutritional compounds, contributed to the changes in root biomass, which was previously noticed (Lekberg and Helgason, 2018). The other growth parameters of plantlets (i.e., shoot and root length, shoot fresh weight) remained unchanged upon mycorrhization. Furthermore, it is also possible that raised content of chlorophyll in leaves of healthy mycorrhizal potato plantlets could be associated with increased photosynthetic rate, as it was demonstrated for potato cv. Marfona (Sipahioglu et al., 2009) and other hosts, e.g., maize (Zare-Maivan et al., 2017), cucumber (Chen S. et al., 2017), or pepper (Beltrano et al., 2013).

Positive effects of mycorrhiza described above were masked in the presence of PVY, thus growth capacity of virus-positive *R. irregularis*-potato plantlets did not differ from the control ones. Nevertheless, mycorrhizal fungi strongly influenced the endogenous level of H_2_O_2_ in both healthy and PVY-infected potato plants. In the absence of viral pathogen, colonization of potato cv. Pirol roots with *R. irregularis* slightly (but significantly) increased H_2_O_2_ level in plant root but not shoot. The generation of ROS in plants, as a response to the mycorrhizal colonization of roots, was previously described (Puppo et al., 2013; Nath et al., 2016; Kapoor and Singh, 2017). ROS acting as long distance signal molecules play an important role during plant adaptation to biotic stress (Sewelam et al., 2016). Interestingly, our study revealed prolonged maintenance of elevated H_2_O_2_ in mycorrhizal potato plantlets, which is in line with the previously described results (Thiem et al., 2014), however the basis of this observation is unclear and requires further consideration. Nevertheless, Hause and Fester (2005) demonstrated that H_2_O_2_ is produced in arbuscules and the use of ROS scavengers (e.g., ascorbic acid and salicylhydroxamic acid) reduces both H_2_O_2_ level and mycorrhizal development (Kapoor and Singh, 2017).

Mycorrhizal fungi exerted the most pronounced effect in PVY-infected potato plants, causing considerable reduction of endogenous H_2_O_2_ content. This effect was stronger in plant shoot where H_2_O_2_ concentration was restored down to the control level, thus suggesting the protective role of mycorrhiza against PVY-induced oxidative stress. However, in the roots of mycorrhizal plantlets, the level of H_2_O_2_ was only partially lowered. The maintenance of oxidative stress in plant root means that this organ was the most affected by the interaction between host, PVY and AMF. As it was discussed above, typical plant reaction to pathogen invasion involves increased production of H_2_O_2_. In our experiment mycorrhiza alleviated stress caused by PVY. The result is contradictive to study by Sipahioglu et al. (2009) suggesting that mycorrhiza enhances the negative impact of the virus and exacerbates disease symptoms in sensitive potato cv. Marfona. This discrepancy may be related to the various characteristics of potato cultivars used in both experiments (tolerant vs. sensitive), since virus behavior may differ depending on the host genotype (Valkonen, 2015; Davie et al., 2017). Nevertheless, the investigation by Maffei et al. (2014), examining different experimental biosystem consisting of tomato, Tomato yellow leaf curl Sardinia virus (TYLCSV) and *Funneliformis mosseae*, indicated attenuation of viral disease symptoms upon symbiosis. More recently, Wang et al. (2018) showed that mycorrhization of tomato affected by *Cladosporium fulvum* (pathogenic mold) caused significant increase in activities of both superoxide dismutase and peroxidase, which correlated with decrease in H_2_O_2_ level. We suspect that similar biological processes may explain reduced concentration of H_2_O_2_ in virus-positive potato host, however molecular studies are necessary to confirm this presumption.

## Conclusion

Our study demonstrated that infection with PVY^N:O^-T1, although asymptomatic, negatively affected vegetative growth of the Pirol cultivar. Furthermore, the virus induced stress response in plants. *R. irregularis* inoculation had slightly positive effect on plantlets’ growth parameters, however mycorrhizal benefits were inhibited by PVY. The processes that cause the effects of PVY infection to be more pronounced over the mycorrhizal benefits are not identified yet.

Interestingly, mycorrhiza modulated plant-pathogen interaction. The effect of PVY infection in potato can be alleviated and masked by mycorrhizal symbiosis. In consequence of this result the molecular mechanism underlying this biotic interactions and the practical consequences for field observations in potato breeding need to be analyzed.

## Data Availability Statement

The datasets generated for this study can be found in the sequences were deposited in GenBank under accession numbers MK455818 and MK455819.

## Author Contributions

ED-S was responsible for the original draft preparation, review and editing of the manuscript, multiplex RT-PCR, sequencing, data annotation, virus identification, phylogenetic tree, ELISA, and statistical analyses. AK worked on the *in vitro* culture of AMF and mycorrhiza staining. AT worked on the *in vitro* culture of potato and analysis of plant growth parameters. AS-J analyzed the hydrogen peroxide. CB and LM participated in the review and the editing of the manuscript. KH conceptualized the study, supervised, reviewed, and edited the manuscript, and was responsible for the funding acquisition. All authors read and approved the final manuscript.

## Conflict of Interest

LM was employed by the company INOQ GmbH, Schnega, Germany. The remaining authors declare that the research was conducted in the absence of any commercial or financial relationships that could be construed as a potential conflict of interest.
