# Supplementary material for: Arbuscular Mycorrhiza Changes the Impact of Potato Virus Y on Growth and Stress Tolerance of Solanum tuberosum L. in vitro
Source: Front Microbiol. 2020 Jan 15;10:2971. doi: 10.3389/fmicb.2019.02971 (PMC6974554; doi:10.3389/fmicb.2019.02971)
Supplement: Supplementary file 1 [file Table_1.docx]

**Supplementary Material**

Table 1. Two-way ANOVA showing the effect of PVY-mycorrhiza interaction on growth parameters (shoot length and fresh weight, root length and fresh weight) in plantlets of potato cv. Pirol.

|  | **Shoot length (mm)** | | | | **Shoot FW (mg)** | | | | **Root length (mm)** | | | | **Root FW (mg)** | | | |
| --- | --- | --- | --- | --- | --- | --- | --- | --- | --- | --- | --- | --- | --- | --- | --- | --- |
|  | MS effect | F | | P | MS effect | F | | P | MS effect | F | | P | MS effect | F | | P |
| (A) *S. tuberosum*  Pirol^PVY-/PVY+^ | 11034.2 | 26.6326 | | **0.0000*** | 19236.5 | 10.8586 | | **0.0024*** | 43143.2 | 122.5548 | | **0.0000*** | 15731.04 | 57.0204 | | **0.0000*** |
| (B) Inoculation | 319.4 | 0.7709 | | 0.3863 | 440.7 | 0.2487 | | 0.6213 | 1406.2 | 3.9946 | | 0.0534 | 2989.96 | 10.8377 | | **0.0026*** |
| (A) x (B) | 482.8 | 1.1654 | | 0.2882 | 36.9 | 0.0208 | | 0.8861 | 552.8 | 1.5704 | | 0.2187 | 1933.01 | 7.0066 | | 0.0128 |
| Error | 414.3 |  | |  | 1771.5 |  | |  | 352.0 |  | |  | 275.88 |  | |  |
| The effect of PVY  The effect of AMF | PIROL^PVY-^  PIROL^PVY+^    PIROL^PVY-^  PIROL^PVY-^ + Ri  PIROL^PVY+^  PIROL^PVY+^+ Ri | | **112.64 a**  **83.80 b**  112.64 a  127.00 a  83.80 b  83.30 b | | PIROL^PVY-^  PIROL^PVY+^    PIROL^PVY-^  PIROL^PVY-^ + Ri  PIROL^PVY+^  PIROL^PVY+^+ Ri | | 172.75 a  128.96 ab  172.75 a  167.83 ab  128.96 ab  120.02 b | | PIROL^PVY-^  PIROL^PVY+^    PIROL^PVY-^  PIROL^PVY-^ + Ri  PIROL^PVY+^  PIROL^PVY+^+ Ri | | **87.40 a**  **27.56 b**  87.40 a  107.22 a  27.56 b  32.10 b | | PIROL^PVY-^  PIROL^PVY+^    PIROL^PVY-^  PIROL^PVY-^ + Ri  PIROL^PVY+^  PIROL^PVY+^+ Ri | | **47.78 a**  **18.66 b**  **47.78 a**  **83.04 c**  18.66 b  22.49 b | |

*p≤0.05; MS mean square; F ratio of MS (effect) to MS (error). Statistically significant differences are given in bold.
