# Supplementary material for: Arbuscular Mycorrhiza Changes the Impact of Potato Virus Y on Growth and Stress Tolerance of Solanum tuberosum L. in vitro
Source: Front Microbiol. 2020 Jan 15;10:2971. doi: 10.3389/fmicb.2019.02971 (PMC6974554; doi:10.3389/fmicb.2019.02971)
Supplement: Supplementary file 2 [file Table_2.docx]

**Supplementary Material**

Table 2. Two-way ANOVA showing the effect of PVY-mycorrhiza interaction on chlorophyll content and stress response (H_2_O_2_ level in shoot and root) in plantlets of potato cv. Pirol.

|  | **Chlorophyll (µg g^-1^ FW)** | | | | **H_2_O_2_ in shoot (µmol g^-1^ FW)** | | | | **H_2_O_2_ in root (µmol g^-1^ FW)** | | | |
| --- | --- | --- | --- | --- | --- | --- | --- | --- | --- | --- | --- | --- |
|  | MS effect | F | | P | MS effect | F | | P | MS effect | F | | P |
| (A) *S. tuberosum*  Pirol^PVY-/PVY+^ | 29319 | 10.972 | | **0.0020*** | 0.31692 | 132.59 | | **0.0000*** | 4.0252 | 268.35 | | **0.0000*** |
| (B) Inoculation | 65430 | 24.485 | | **0.0000*** | 0.50405 | 210.90 | | **0.0000*** | 0.4447 | 29.65 | | **0.0006*** |
| (A) x (B) | 62019 | 23.208 | | **0.0000*** | 0.39762 | 166.37 | | **0.0000*** | 1.4630 | 97.53 | | **0.0000*** |
| Error | 2672 |  | |  | 0.00239 |  | |  | 0.015 |  | |  |
| The effect of PVY  The effect of AMF | PIROL^PVY-^  PIROL^PVY+^    PIROL^PVY-^  PIROL^PVY-^ + Ri  PIROL^PVY+^  PIROL^PVY+^+ Ri | | 407.04 a  431.30 a  **407.04 a**  **564.45 b**  431.30 a  433.41 a | | PIROL^PVY-^  PIROL^PVY+^    PIROL^PVY-^  PIROL^PVY-^ + Ri  PIROL^PVY+^  PIROL^PVY+^+ Ri | | **0.81 a**  **1.51 b**  0.81 a  0.79 a  **1.51 b**  **0.78 a** | | PIROL^PVY-^  PIROL^PVY+^    PIROL^PVY-^  PIROL^PVY-^ + Ri  PIROL^PVY+^  PIROL^PVY+^+ Ri | | **0.87 a**  **2.73 b**  **0.87 a**  **1.19 c**  **2.73 b**  **1.65 d** | |

*p≤0.05; MS mean square; F ratio of MS (effect) to MS (error). Statistically significant differences are given in bold.
